# The complete chloroplast genome of *Erigeron sumatrensis* (Asteraceae): genomic features and phylogenetic implications

**DOI:** 10.1080/23802359.2026.2722794

**Published:** 2026-10-15

**Authors:** Yu Liu, Xin Wei, Jing Li, Jinghui Gao, Xiaowen Li, Jinwang Wang

**Affiliations:** Wenzhou Key Laboratory of Resource Plant Innovation and Utilization, Zhejiang Institute of Subtropical Crops, Zhejiang Academy of Agricultural Sciences, Wenzhou, Zhejiang, China

**Keywords:** *Erigeron sumatrensis*, chloroplast genome, Invasive weed, phylogeny

## Abstract

*Erigeron sumatrensis* Retz. (Asteraceae) is an annual herb native to South America that has become a noxious invasive weed in tropical and subtropical regions worldwide. Here, we report the first complete chloroplast genome of this plant species. The chloroplast genome is 152,974 bp in length and exhibits a typical quadripartite structure, consisting of a large single-copy (LSC) region of 84,615 bp, a small single-copy (SSC) region of 18,335 bp, and a pair of inverted repeat (IR) regions of 25,012 bp each. The overall GC content is 37.16%. The genome encodes 133 genes, including 88 protein-coding genes, 37 transfer RNA (tRNA) genes, and 8 ribosomal RNA (rRNA) genes. Phylogenetic analysis based on 29 chloroplast genomes indicated its close affinity to *E. rupicola and E. fernandezianus* within *Erigeron*. This study provides an important genomic resource for species identification, population genetics, and research on the invasion mechanism of *E. sumatrensis*.

## Introduction

*Erigeron sumatrensis* Retz. (syn. *Conyza sumatrensis* (Retz.) E. Walker 1788) is an erect annual herb, typically 60–150 cm tall, with branched stems and alternate, oblanceolate to narrowly elliptic leaves. The capitula are small, 5–7 mm in diameter, arranged in terminal panicles, bearing numerous white to pale purple ray florets and yellow disk florets. Morphologically, *E. sumatrensis* closely resembles the congeneric species *E. bonariensis*; however, it can be distinguished by its more robust habit, slightly larger and more densely clustered capitula, and ray florets with a subtle purplish tinge. This species is listed as a level‑1 malignant invasive plant (Yan et al. 2014). In areas such as the Three Gorges River Valley in China, it has significantly altered the interspecific associations of native plant communities, reduced community stability, and posed serious threats to agricultural production and biodiversity (Zhou et al. 2024). *E. sumatrensis* is morphologically highly similar to the congeneric species *E. bonariensis* and *E. canadensis*, making accurate identification difficult when relying solely on subtle traits such as inflorescence size and ray floret color, especially during the vegetative stage where misidentification rates are very high. This difficulty directly hampers the implementation of effective invasion control strategies. However, the complete chloroplast genome of *E. sumatrensis* has not yet been reported, limiting evolutionary studies and molecular marker development for this species.

Owing to its maternal inheritance, structural conservation, and moderate sequence variation, the chloroplast genome has become an ideal tool for plant phylogenetic reconstruction, species identification, and evolutionary studies (Abdullah et al. 2025). In this study, we sequenced, assembled, and annotated the complete chloroplast genome of *E. sumatrensis* for the first time and constructed a phylogenetic tree based on chloroplast genomes of 27 *Erigeron* species and 2 *Solidago* species, aiming to clarify the systematic position of this species and provide a genomic foundation for identification of closely related species and invasion biology research.

## Materials and methods

Fresh leaves were collected from three individual plants growing at the field experiment base of the Zhejiang Institute of Subtropical Crops, Zhejiang Province, China (27°57’N, 120°31’E), with 2–3 young and healthy leaves taken from each plant. The leaf samples from the three individuals were pooled for DNA extraction to obtain a representative chloroplast genome sequence (Figure 1). The plant material was identified by the corresponding author (contact: Jinwang Wang, kingwwang@163.com), and a voucher specimen (specimen number: SMBSC2025-01) has been deposited at the herbarium of the Zhejiang Institute of Subtropical Crops. The collected leaf samples were immediately frozen in liquid nitrogen and stored at −80 °C until DNA extraction. Total genomic DNA was extracted using a modified cetyltrimethylammonium bromide (CTAB) method (Doyle and Doyle 1987). DNA concentration and purity were assessed using a NanoDrop 2000 spectrophotometer and 1% agarose gel electrophoresis.

**Figure 1.**
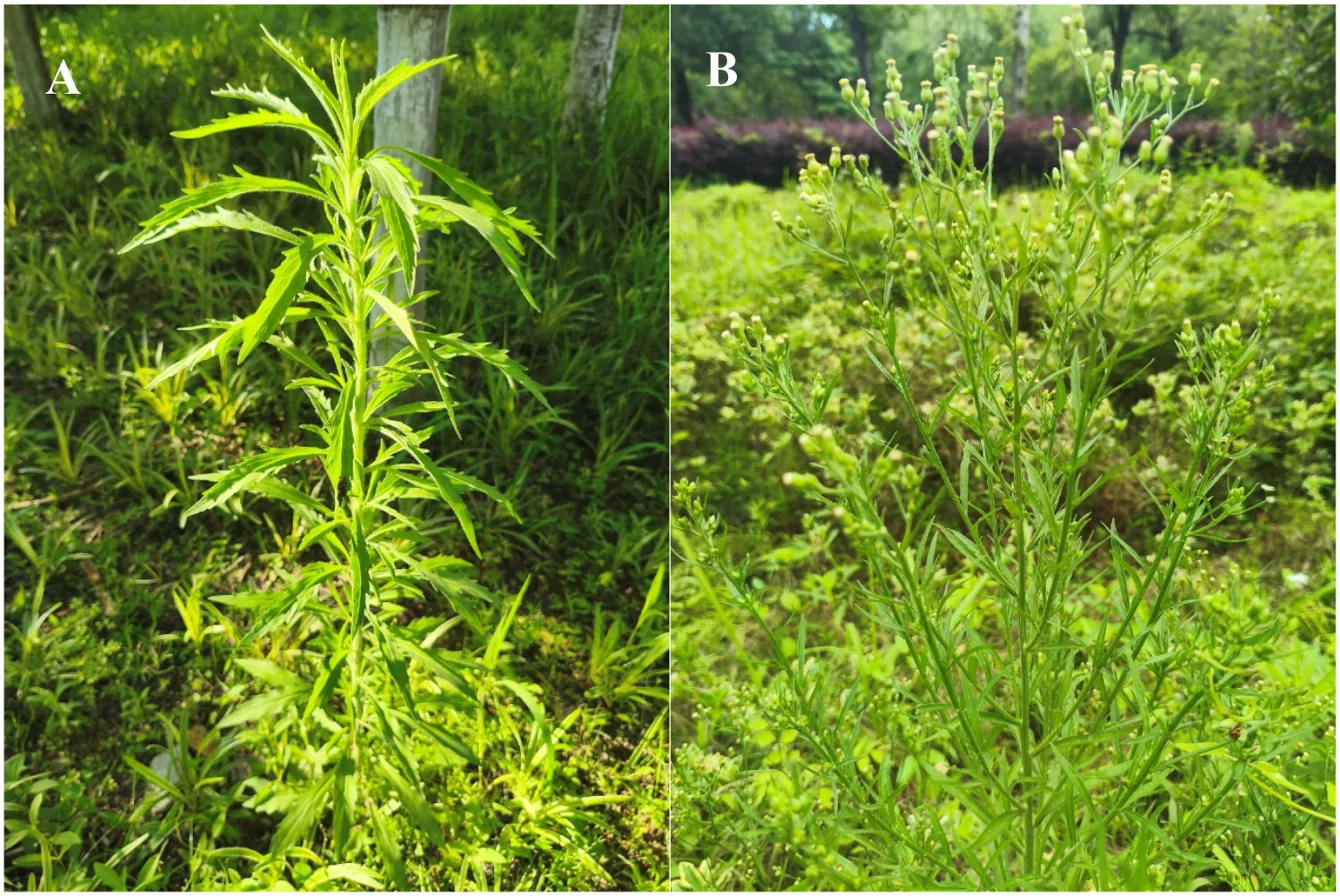
Plant morphology of *E. sumatrensis*. (A) Whole plant habit. (B) Close-up of capitulum showing white to pale purple ray florets and yellow disk florets. Photograph taken by ouhai in wenzhou city, Zhejiang province and was taken by the authors.

High-quality genomic DNA was sheared into fragments of approximately 300–500 bp using an ultrasonic processor. DNA libraries were constructed following the manufacturer’s protocol for the BGISEQ-T7 platform. Briefly, end-repair, A-tailing, and adapter ligation were performed, followed by PCR amplification and library purification. Sequencing was conducted on the BGISEQ-T7 platform using a paired-end 150-bp read length.

Raw sequencing reads were quality-filtered and adapter-trimmed using Fastp v1.0.1 (Chen et al. 2018). Reads with >5% ambiguous bases (N), a quality score Q20 below 20%, or length <50 bp were removed. Clean reads were mapped to a customized chloroplast genome database using Minimap2 v2.30 (Li 2021). Chloroplast genome assembly was performed using NOVOPlasty v4.3.1 (Dierckxsens et al. 2017) with the chloroplast genome of *E. acris* (GenBank accession: BK070975) as the reference. The initial assembly was visualized and manually checked using Geneious v9.1.8 (Kearse et al. 2012). The complete circular chloroplast genome sequence was deposited in GenBank under the accession number PZ435482.

Gene annotation was performed using the online tool GeSeq (Tillich et al. 2017) for protein-coding genes and ribosomal RNA genes, and tRNAscan-SE v2.0 (Chan et al. 2021) for transfer RNA genes. Annotations were manually curated to ensure accurate start and stop codon positions and intron boundary assignments. A circular gene map of the chloroplast genome was generated using OGDRAW v1.3.1 (Greiner et al. 2019). A graphical map of cis-splicing genes and the trans-splicing gene, was generated using CPGView (Liu et al. 2023).

Two species (*Solidago canadensis* and *S. decurrens*) were used as outgroups, and a total of 27 chloroplast genomes of Erigeron (including *E. sumatrensis*) were selected for phylogenetic tree construction. Phylogenetic analysis was performed based on whole-genome sequence alignment. The sequences used in this study were downloaded from NCBI GenBank. The complete chloroplast genomes of all 29 species (including the outgroups) were aligned using MAFFT v7.0 with the –auto mode, starting from the same genomic position (the LSC/IRb junction) (Katoh and Standley 2013). Poorly aligned regions and gaps were manually inspected and removed using Geneious v9.1.8 (Kearse et al. 2012). For the inverted repeat (IR) regions, we retained the full-length sequences as recommended for phylogenomic analyses, and both IR copies were included in the alignment. Then, FastTree v2.1.10 was used to build the maximum likelihood (ML) evolutionary tree under the GTR+G substitution model, which was determined as the best-fit model by ModelFinder (Price et al. 2010). A preliminary analysis using IQ-TREE (with ModelFinder) yielded a topology consistent with that obtained from FastTree, confirming the robustness of our phylogenetic results.

## Results

The complete chloroplast genome of *E. sumatrensis* is a circular molecule of 152,974 bp in length (Figure S1). The genome exhibits the typical quadripartite structure of angiosperm chloroplast genomes, consisting of a large single-copy (LSC) region of 84,615 bp, a small single-copy (SSC) region of 18,335 bp, and a pair of inverted repeat (IR) regions of 25,012 bp each (Figure 2). The overall GC content of the genome is 37.16%. The GC content is unevenly distributed among regions: the IR regions show the highest GC content (42.98%), while the LSC and SSC regions exhibit lower GC contents (35.07% and 30.96%, respectively).

**Figure 2.**
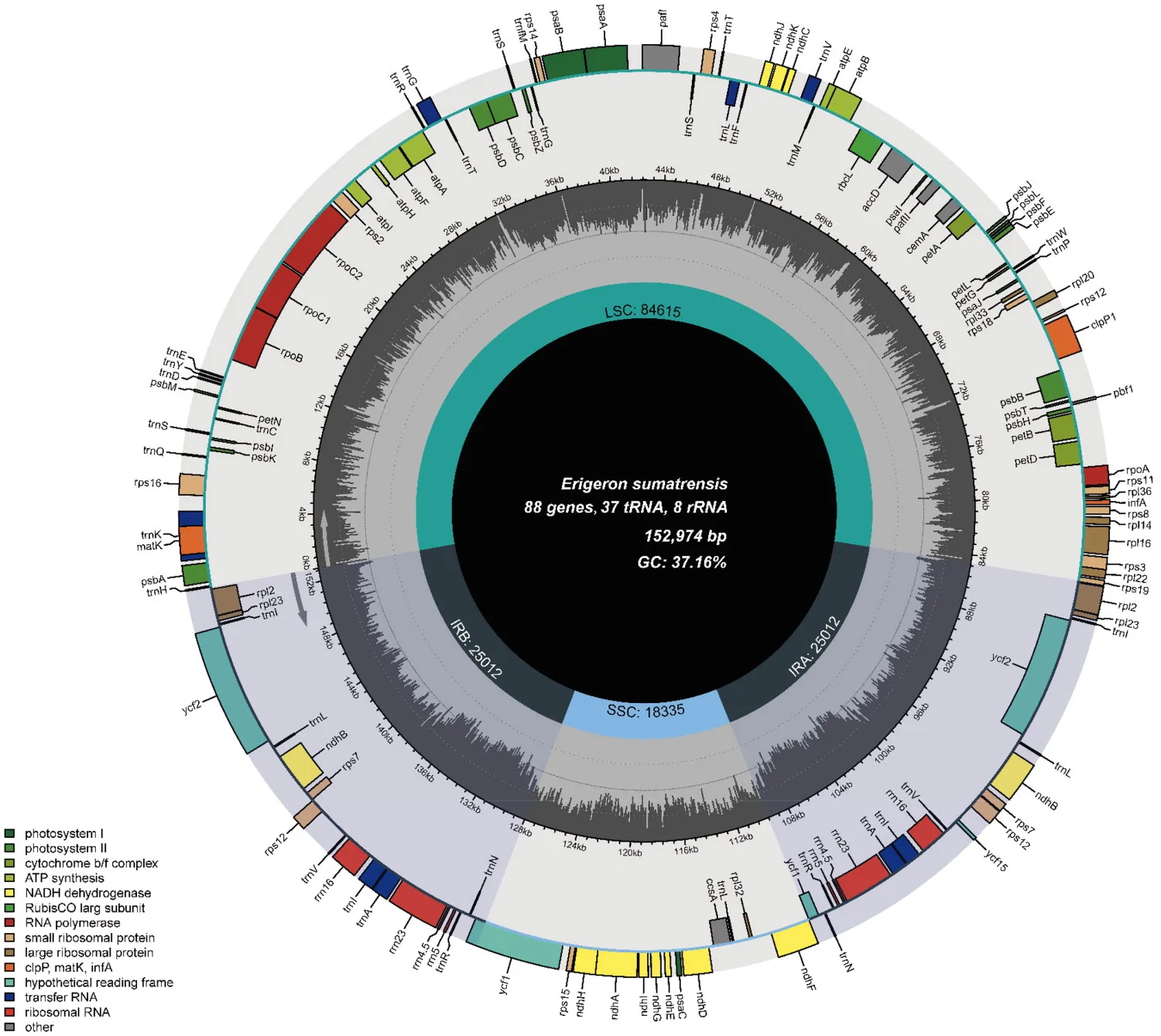
Gene map of the *E. sumatrensis* cp genome. Note: the forward-coding genes are located on the outside of the circle, and the reverse-coding genes are located on the inside of the circle. The color coding indicates gene function. In the inner circle, dark grey represents the GC content and light grey represents the AT content.

The chloroplast genome encodes a total of 133 genes, comprising 88 protein-coding genes, 37 transfer RNA (tRNA) genes, and 8 ribosomal RNA (rRNA) genes (Table S1). Among the 88 protein-coding genes, nine genes contain a single intron (*ndhA*, *ndhB*, *atpF*, *petB*, *petD*, *rpoC1*, *rpl2*, *rpl16*, *rps16*), and three genes (*clpP1, rps12* and *pafI*) contain two introns, consistent with the typical intron distribution pattern observed in Asteraceae chloroplast genomes (Figure S2 and Figure S3). The two copies of the IR region carry duplicated genes, including four rRNA genes (*rrn4.5*, *rrn5*, *rrn16*, *rrn23*), seven tRNA genes, and several protein-coding genes (*rpl2*, *rpl23*, *rps7*, *rps12*, *rps19*, *ycf1*, *ycf2*, *ycf15*). The inverted repeats are separated by the LSC and SSC regions, as is typical for angiosperm chloroplast genomes.

Based on the maximum-likelihood phylogenetic tree constructed from 29 chloroplast genome sequences, the phylogenetic relationships within the genus *Erigeron* are clearly resolved (Figure 3). Using *S. canadensis* and *S. decurrens* as outgroups, all *Erigeron* and former *Conyza* species formed a monophyletic clade, which was further divided into two major subclades, and these two subclades are sister to each other. Most internal nodes within these subclades have high bootstrap support values (49% to 100%). *E. sumatrensis* together with *E. rupicola* and *E. fernandezianus* formed a well-supported subclade (bootstrap support values ranging from 99% to 100%).

**Figure 3.**
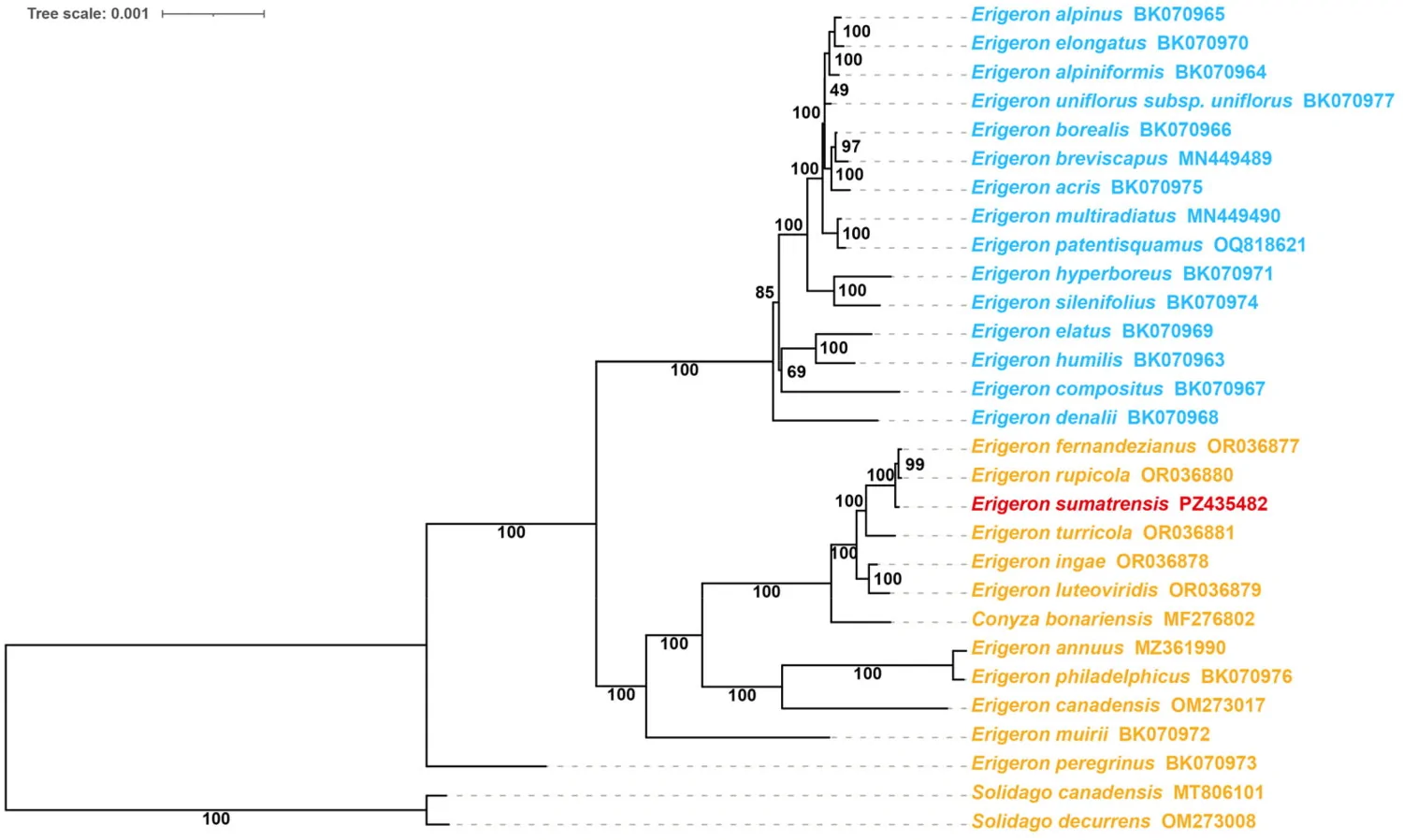
Maximum likelihood (ML) phylogenetic tree based on the complete chloroplast genomes of 27 *erigeron* species and two *solidago* species as outgroups. The newly reported species *E. sumatrensis* is highlighted in red bold. The two major subclades are distinguished by background shading: light blue for subclade I and light orange for subclade II. Bootstrap support values are shown at nodes. The following sequences are used: *Erigeron humilis* BK070963 (Abdullah et al. 2025); *erigeron alpiniformis* BK070964 (Abdullah et al. 2025); *erigeron alpinus* BK070965 (Abdullah et al. 2025); *erigeron borealis* BK070966 (Abdullah et al. 2025); *erigeron compositus* BK070967 (Abdullah et al. 2025); *erigeron denalii* BK070968 (Abdullah et al. 2025); *erigeron elatus* BK070969 (Abdullah et al. 2025); *erigeron elongatus* BK070970 (Abdullah et al. 2025); *erigeron hyperboreus* BK070971 (Abdullah et al. 2025); *erigeron muirii* BK070972 (Abdullah et al. 2025); *erigeron peregrinus* BK070973 (Abdullah et al. 2025); *erigeron silenifolius* BK070974 (Abdullah et al. 2025); *erigeron acris* BK070975 (Abdullah et al. 2025); *erigeron philadelphicus* BK070976 (Abdullah et al. 2025); *erigeron uniflorus* subsp. *Uniflorus* BK070977 (Abdullah et al. 2025); *erigeron canadensis* MT806101 (Sang and Jongsun 2023); *conyza bonariensis* MF276802 (Hereward et al. 2017); *erigeron breviscapus* MN449489 (Li et al. 2019); *erigeron multiradiatus* MN449490 (Li et al. 2019); *erigeron annuus* MZ361990 (Zhou et al. 2022); *erigeron patentisquamus* OQ818621; *erigeron fernandezianus* OR036877 (Kim et al. 2024); *erigeron ingae* OR036878 (Kim et al. 2024); *erigeron luteoviridis* OR036879 (Kim et al. 2024); *erigeron rupicola* OR036880 (Kim et al. 2024); *erigeron turricola* OR036881 (Kim et al. 2024); *solidago canadensis* OM273017 (Tao et al. 2024); *solidago decurrens* OM273008 (Tao et al. 2024); *erigeron sumatrensi*s PZ435482.

## Discussion and conclusion

The complete chloroplast genome of *E. sumatrensis* reported in this study exhibits the typical quadripartite structure and gene content characteristic of Asteraceae chloroplast genomes. The genome size (152,974 bp) falls within the range reported for other *Erigeron* species (152,108 to 153,430 bp). Gene content analysis revealed 133 genes, consisting of 88 protein-coding genes, 37 tRNAs, and 8 rRNAs. As shown in Table 1, the chloroplast genomic features of *E. sumatrensis* are largely congruent with other *Erigeron* species. The genome sizes of the examined taxa span 152,108 bp (*E. elatus*) to 153,430 bp (*E. muirii*). Gene repertoires differ moderately among species, with total gene numbers ranging from 128 to 133. The GC content shows minor interspecific divergence, ranging from 37.00% to 37.30%. The elevated GC content observed in the IR regions (42.98% versus 35.07% in LSC and 30.96% in SSC) is a conserved feature across angiosperms, attributable to the presence of rRNA genes that require stable secondary structures enriched in G-C pairs. The presence of two introns in *clpP1*, *pafI*, and *rps12* is particularly noteworthy, as these introns are known to be evolutionarily variable across Asteraceae. For instance, previous studies have documented length and sequence polymorphisms in the *rps12* intron, which have been effectively used to resolve phylogenetic relationships within Astereae (Liu et al. 2026). Similarly, variations in the *clpP1* intron have been widely applied in genus-level phylogenetic analyses of Asteraceae (Huang et al. 2025). Our observation that *pafI* contains two introns is consistent with reports from other Asteraceae lineages, further supporting the potential utility of these intronic regions as informative molecular markers for phylogenetic inference at shallower taxonomic levels.

**Table 1.** Comparison of chloroplast genome features among *E. sumatrensis* and representative *Erigeron* species.

Species	Genome size (bp)	LSC (bp)	SSC (bp)	IR (bp)	GC content (%)	Total genes	Protein-coding genes	tRNAs	rRNAs	References
*E. sumatrensis*	152,974	84,615	18,335	25,012	37.16	133	88	37	8	This study
*E. alpiniformis*	152258	84,756	18,106	24,698	37.18	128	84	36	8	Abdullah et al. 2025
*E. alpinus*	152433	84,912	18,125	24,698	37.14	128	84	36	8	Abdullah et al. 2025
*E. borealis*	152305	84,795	18,116	24,697	37.17	128	84	36	8	Abdullah et al. 2025
*E. compositus*	152421	84,840	18,185	24,698	37.14	128	84	36	8	Abdullah et al. 2025
*E. denalii*	152270	84,730	18,150	24,695	37.13	128	84	36	8	Abdullah et al. 2025
*E. elatus*	152108	84,611	18,097	24,700	37.19	128	84	36	8	Abdullah et al. 2025
*E. elongatus*	152457	84,941	18,120	24,698	37.14	128	84	36	8	Abdullah et al. 2025
*E. hyperboreus*	152788	85,191	18,185	24,706	37.08	128	84	36	8	Abdullah et al. 2025
*E. muirii*	153430	84,856	18,530	25,022	37.09	128	84	36	8	Abdullah et al. 2025
*E. peregrinus*	152631	84,250	18,273	25,054	37.30	128	84	36	8	Abdullah et al. 2025
*E. silenifolius*	152420	84,797	18,215	24,704	37.09	128	84	36	8	Abdullah et al. 2025
*E. acris*	152438	84,931	18,111	24,698	37.14	128	84	36	8	Abdullah et al. 2025
*E. philadelphicus*	153151	84,700	18,413	25,019	37.07	128	84	36	8	Abdullah et al. 2025
*E. uniflorus*	152309	84,779	18,134	24,698	37.17	128	84	36	8	Abdullah et al. 2025
*E. canadensis*	152,767	84,317	18,446	25,004	37.10	133	88	37	8	Sang and Jongsun 2023
*E. breviscapus*	152,367	84,881	18,102	24,692	37.10	129	84	37	8	Li et al. 2019
*E. multiradiatus*	152,281	84,789	18,110	24,691	37.20	129	84	37	8	Li et al. 2019
*E. annuus*	153,177	84,727	18,412	25,019	37.00	130	87	35	8	Zhou et al. 2022
*E. fernandezianus*	153,031	84,630	25,023	18,355	37.2	130	85	36	8	Kim et al. 2024
*E. ingae*	152,962	84,597	25,012	18,341	37.2	130	85	36	8	Kim et al. 2024
*E. luteoviridi*	152,878	84,605	25,027	18,219	37.2	130	85	36	8	Kim et al. 2024
*E. rupicola*	152,993	84,635	25,012	18,334	37.2	131	85	36	8	Kim et al. 2024
*E. turricola*	152,740	84,864	24,770	18,336	37.2	130	85	36	8	Kim et al. 2024

Phylogenetic analysis based on 29 chloroplast genome sequences reveals an important evolutionary pattern: all former *Conyza* species (*C. bonariensis* and *E. canadensis*), together with *E. sumatrensis* and others, are embedded within a well-supported clade inside the genus *Erigeron*, rather than forming an outgroup independent of *Erigeron*. Unlike previous morphological classifications, the phylogenetic analysis based on complete chloroplast genomes in this study clearly shows that the former *Conyza* species do not constitute an independent clade but rather represent a specialized lineage within *Erigeron* (Nesom 2008; Noyes 2000).

The chloroplast genome of *E. sumatrensis* obtained in this study provides important foundational data for the future development of species-specific molecular markers. Combined with the clear distinction between *E. sumatrensis* and its closely related species in the phylogenetic tree, primer pairs could be designed for rapid and accurate identification of *E. sumatrensis*, thereby providing technical support for early warning and precise management of this invasive plant.

## Supplementary Material

Supplemental.docx

## Data Availability

The genome sequence data that support the findings of this study are openly available in GenBank of NCBI at (https://www.ncbi.nlm.nih.gov/) under the accession no. PZ435482. The associated BioProject, SRA, and Bio-Sample numbers are PRJNA1466718, SRR38624543, and SAMN60150253, respectively.
