# Supplementary material for: The complete chloroplast genome of Erigeron sumatrensis (Asteraceae): genomic features and phylogenetic implications
Source: Mitochondrial DNA B Resour. 2026 Oct 15;11(11):1333–8. doi: 10.1080/23802359.2026.2722794 (PMC13674646; doi:10.1080/23802359.2026.2722794)
Supplement: Supplementary file 1 [file TMDN_A_2722794_SM1298.docx]

The complete chloroplast genome of *Erigeron sumatrensis* (Asteraceae): genomic features and phylogenetic implications

Yu Liu^1^, Xin Wei^1^, Jing Li^1^, Jinghui Gao^1^, Xiaowen Li^1^, Jinwang Wang ^1*^

1 Wenzhou Key Laboratory of Resource Plant Innovation and Utilization, Zhejiang Institute of Subtropical Crops, Zhejiang Academy of Agricultural Sciences, Wenzhou 325005, Zhejiang, China

For correspondence email: kingwwang@163.com (JW.W.)

Yu Liu ([liuyu@zaas.ac.cn](mailto:liuyu@zaas.ac.cn))

Xin Wei ([wiex@.zaas.ac.cn](mailto:wiex@.zaas.ac.cn))

Jing Li (lijing@csuft.edu.cn

Jinghui Gao ([gjh630427233@163.com](mailto:gjh630427233@163.com))

Xiaowen Li ([linxw@zaas.ac.cn](mailto:linxw@zaas.ac.cn))

Jinwang Wang (kingwwang@163.com)

**Figure S1** Generating Sequencing Depth and Coverage Map for *E. sumatrensis* chloroplast genome. X and Y axis present nucleotide position of *E. sumatrensis* chloroplast genome and read mapping depth, respectively. The total genomic length =152,974 bp, average depth = 1046.3 x.

**Figure S2** Structural organization of cis-splicing genes in the Erigeron sumatrensis chloroplast genome. Exons are represented by filled boxes, and introns by horizontal lines connecting exons. Gene names are shown in italics. The following genes with a single intron are presented: *rps16*, *rpoC1*, *atpF*, *petB*, *petD*, *rpl16*, *rpl2*, *ndhB*, and *ndhA*. The positions and relative lengths of exons and introns are drawn to scale based on the genome annotation. This schematic illustrates the splicing pattern of each cis-spliced gene, in which all exons are located within the same transcriptional unit and the intron is removed by a single splicing event.

**Figure S3** Structural organization of the trans-splicing gene *rps12* in the Erigeron sumatrensis chloroplast genome. Exons are represented by filled boxes, and introns by horizontal lines. Gene names are shown in italics. The *rps12* gene is a trans-spliced gene with its exons located in two separate genomic regions: exon 1 (5′ exon) is situated in the large single-copy (LSC) region, while exons 2 and 3 (3′ exons) are duplicated in the two inverted repeat (IR) regions. The intron between exons 1 and 2 is removed via a trans-splicing mechanism that joins transcripts from different genomic locations. This structural arrangement is characteristic of *rps12* in most angiosperm chloroplast genomes.

**Table** S**1**. Chloroplast genome-encoded gene types and functional classification of *E. sumatrensis*.

| Category | Cene Group | Cene Name |
| --- | --- | --- |
| Photosynthesis | Subunits of photosystem I | *psaA, psaB, psaC, psaI, psaJ, pafI**, pafII* |
|  | Subunits of photosystem II | *psbA, psbB, psbC, psbD, psbE, psbF, psbH, psbI, psbJ, psbK, psbL, psbM, psbT, psbZ, pbf1* |
|  | Subunits of cytochrome b/f complex | *petA, petB*, petD*, petG, petL, petN* |
|  | Subunits of ATP synthase | *atpA, atpB, atpE, atpF*, atpH, atpI* |
|  | Subunits of NADH dehydrogenase | *ndhA*, ndhB (×2) *, ndhC, ndhD, ndhE, ndhF, ndhG, ndhH, ndhI, ndhJ, ndhK* |
|  | Subunit of rubisco | *rbcL* |
| Self-replication | RNA polymerase | *rpoA, rpoB, rpoC1*, rpoC2* |
|  | Large subunit of ribosomal proteins | *rpl2 (×2) *, rpl14, rpl16*, rpl20, rpl22, rpl23 (× 2), rpl32, rpl33, rpl36* |
|  | Small subunit of ribosomal proteins | *rps2, rps3, rps4, rps7 (×2), rps8, rps11, rps12 (× 2) **, rps14, rps15, rps16*, rps18, rps19 (× 2)* |
|  | Ribosomal RNAs | *rrn4.5 (×2), rrn5 (× 2), rrn16 (× 2), rrn23 (× 2)* |
|  | Transfer RNAs | *trnH-GUG, trnK-UUU*, trnQ-UUG, trnS-GCU, trnC-GCA, trnD-GUC, trnY-GUA, trnE-UUC, trnR-UCU, trnG-UCC*, trnT-GGU○1, trnS-UGA, trnG-GCC, trnfM-CAU, trnS-GGA, trnT-UGU, trnL-UAA*, trnF-GAA, trnV-UAC*, trnM-CAU, trnW-CCA, trnP-UGG, trnI-CAU(×2), trnL-CAA(×2), trnV-GAC(×2), trnI-GAU* (×2), trnA-UGC* (×2), trnR-ACG(×2), trnN-GUU(×2), trnL-UAG* |
| Other genes | Subunit of acetyl-coA-carboxylase | *accD* |
|  | c-type cytochrome synthesis gene | *ccsA* |
|  | Envelop membrane protein | *cemA* |
|  | Protease | *clpP1*** |
|  | Translational initiation factor | *infA* |
|  | Maturase | *matK* |
| Genes of unknown function | Conserved open reading frames | *ycf1 (×2), ycf2 (×2) , ycf15(×2)* |

Note: Gene (×2), Multiple copy gene, the number of copies in parenthesis; Gene *, Gene with two exons; Gene **, Genes containing three exons.
